# The effect of late gestation injectable vitamin A and D supplementation on sow and piglet performance

**DOI:** 10.1093/tas/txaf134

**Published:** 2025-09-30

**Authors:** Samantha R Yankocy, Rachel E Walker, Laura Loughlin, Chelsea Becker, Elizabeth A Hines, Claire Stenhouse

**Affiliations:** Department of Animal Science, College of Agricultural Sciences, Pennsylvania State University, University Park, PA, 16802, United States; Department of Nutritional Sciences, College of Health and Human Development, Pennsylvania State University, University Park, PA, 16802, United States; Department of Animal Science, College of Agricultural Sciences, Pennsylvania State University, University Park, PA, 16802, United States; Department of Animal Science, College of Agricultural Sciences, Pennsylvania State University, University Park, PA, 16802, United States; Department of Animal Science, College of Agricultural Sciences, Pennsylvania State University, University Park, PA, 16802, United States; Department of Animal Science, College of Agricultural Sciences, Pennsylvania State University, University Park, PA, 16802, United States

**Keywords:** immune, retinol, sow, stillborn, vitamin

## Abstract

Vitamin A is an essential nutrient with an important role in animal health across livestock species. Insufficient dietary vitamin A intake is associated with decreased reproductive performance in females as well as impaired growth performance and health of the offspring. The aim of this study was to determine if an intramuscular injection of vitamin A and D (VitAD) given to sows on d 85 of gestation improved the survivability, growth performance, and health of the offspring from birth through the end of the nursery phase. Yorkshire or Yorkshire cross bred sows received either an injection of VitAD (312,000 IU vitamin A, 52,500 IU vitamin D. *n* = 9) or saline control (*n* = 10). At farrowing, birth weights, survival, and umbilical blood was collected on the piglets, and colostrum was collected from sows. Daily weights were taken from d 0 to 4, then weekly weights from d 8 to 63 on all pigs. Milk samples were taken weekly on sows from birth to weaning (d 28), and daily feed consumption for the sows was tracked during the lactation phase. Blood samples were obtained weekly from piglets through weaning (d 28). Subsequent blood samples were taken weekly through the nursery phase (d 28—d 63) from a subset of the original pigs to measure retinol from birth to d 63 and immune parameters from d 28 to d 63. All statistical analyses were run using a mixed linear regression model on SAS (9.4; SAS, Cary, NC). VitAD piglets tended to have a reduced average daily gain (ADG) from d 22 to d 28 (*P *= 0.08), and decreased circulating retinol concentrations on d 15, 22, and 28 (*P *≤ 0.05) compared to control piglets. Fewer stillborn piglets (*P *= 0.01) were present in litters from sows treated with Vit AD compared to control sows. VitAD pigs had an elevated reticulocyte count on d 42 (*P *≤ 0.01) compared to the control pigs. VitAD treated sows consumed less feed on d 15 (*P *≤ 0.05) and had greater milk retinol (*P *≤ 0.01) on d 8 of lactation compared to control sows. These results suggest that the administration of an injection of VitAD to sows on d 85 of gestation might improve offspring survival at birth, as well as decrease sow nutritional consumption requirements during lactation. Additionally, these results help to establish normal immune parameters and circulating retinol in pigs. Importantly, no negative effect of VitAD supplementation on growth rate or immune parameters was observed.

## Teaser

Supplementation of sows with an injection of Vitamin A and D on day 85 of gestation had a limited impact on the performance of the sows at farrowing and on offspring through the end of the weaning phase.

## Introduction

Maternal nutrition is an important determinant of both sow reproductive performance and offspring development and postnatal performance. Inadequate nutrition in breeding sows often manifest as poor performance in sow reproduction and impaired piglet growth (Ball et al 2008). While considerable progress has been made in understanding the macronutrient requirements of sows, the micronutrient requirements, particularly for gestating and lactating sows, remain under investigated and poorly understood. The most recent edition of the Nutrient Requirements of Swine (NRC 2012), cites that the recommendations for most vitamin requirements in the diet have remained unchanged since 1998 (NRC 1998). Marked improvements in sow productivity, driven by significant genetic selection, to increase total number of piglets born live per litter from 8.71 pigs in 1998, to 11.42 pigs in 2023 (USDA 2025) indicate that nutrient requirements should be scrutinized. It could be speculated that this significant improvement in productivity may have altered the physiology of the sow and may have led to alterations in the nutritional requirements for gestating and lactating sows; a concept which warrants further investigation.

Vitamin A in the form of β-carotene has low bioavailability in plant-based feed stuffs utilized for pigs (Braude et al 1941; Ullrey 1972). Furthermore, determination of the appropriate dietary requirement of vitamin A for breeding swine is challenging due to the significant storage of this nutrient in the liver (Parrish et al 1951). Recommended dietary inclusion levels for vitamin A for gestating and lactating swine are 8398 IU and 11,932 IU per day, respectively (NRC 2012). However, it has been suggested that a supplemental injection of vitamin A above NRC recommendations enhances reproductive performance (Coffey and Britt 1993), especially in first and second parity sows (Lindemann et al 2008), with vitamin A supplemented sows having fewer stillborn piglets compared to the control sows.

Retinoic acid, a metabolite of vitamin A, regulates fetal innate immune system development (Randall et al 2008) by controlling inflammatory responses (Oliveira et al 2018). This increase in the adaptive immune response extends to maternal supplementation with vitamin A, which has been suggested to increase circulatory antibody abundance in human infants (Palmer 2011) and serum protein in calves (Salam Abdullah et al 1987). Further, vitamin A deficiency during gestation resulted in impaired retinoic acid signaling during fetal development, which profoundly impaired the ability of the offspring to clear viral infections in mice (Van De Pavert et al 2014). Vitamin A supplementation improves immune development and response (de Azevedo Paiva et al 2010; Van De Pavert et al 2014) and it has been suggested to decrease mortality rate in human children who were supplemented between 6 months and 5 years old (Beaton et al 1994).

To date, few studies have examined the relationship between vitamin A status and the immune response in livestock species. In swine, retinoic acid increased the immune response to *Ascaris suum* infection (Dawson et al 2009). In dairy cows, low vitamin A status was linked to hoof ulcers (Lischer et al 2001). Additionally, studies utilizing mice have suggested that administration of vitamin A decreased pathological changes of the mammary gland infected with *Staphylococcus aureus* (Chew et al 1985). Collectively, these findings support a role of vitamin A in the development and function of the immune system in multiple species. Based on this evidence, it is reasonable to suggest that supplementing vitamin A to sows during late gestation may enhance the development of the fetal immune system and help reduce the incidence and severity of health challenges in both the sows and their offspring.

Recent reports indicate that there are issues with vitamin and mineral deficiencies at all stages of the swine production process. Of greatest interest, a survey of swine across production stages (Greiner et al 2022) indicated that serum levels of vitamins A and D3 were lower than previously published levels across all stages of production, even when fed commercial quality diets. Further, research on the dietary supplementation of vitamin A to sows exposed to porcine endemic diarrhea virus (PEDv) improved passive immunity to the piglets (Amimo et al 2024). Of the vitamin A studies conducted to date that evaluate its effects on piglet survival, most involve sows that receive vitamin A orally or were first fed a diet deficient in vitamin A for an extended period, before receiving vitamin A through an injection or oral supplementation (Parrish et al 1951; Brief and Chew 1985). Few studies have been conducted where sows fed vitamin A sufficient diets with no clinical signs of vitamin A deficiency were given a dose of vitamin A (Coffey and Britt 1993; Pusateri et al 1999; Lindemann et al 2008). The utilization of injectables to supplement vitamin A offers a targeted approach to provide nutritional support to sowsin a more controlled manner to support specific productivity of the sow. However, in the United States, vitamin A is not available commercially to livestock producers in a single compound form. Rather, it is available to producers only in combination with Vitamin E and/or Vitamin D. Supplementation of sows with vitamin D, has been reported to have beneficial to effects on swine reproduction, and due to this evidence, the recommendations for vitamin D requirements was increased from 200 IU to 800 IU (NRC 2012). However, more recent data suggests that further increased supplementation levels of vitamin D may further benefit sows and fetal development. For example, supplementation increases fetal weights by d 90 of gestation (Coffey et al 2012), enhances muscle development in offspring (Hines et al 2013), and elevates concentrations of vitamin D metabolites in colostrum and milk when fed to sows during lactation (Flohr et al 2014). Further, Vitamin D supplementation by injection to gestating sows also improved circulating active metabolite in piglets at birth (Jang et al 2014). Finally, like vitamin A, vitamin D supplementation is associated with improvements in immune response through modification of inflammation and antibody production (Yang and Ma 2021).

In the face of high mortality rates among sows and pigs (Kikuti et al 2023), there is an urgent need for accessible and effective nutritional tools to support pigs through all stages of production. Due to this need, this study aimed to improve the understanding of the effects of a supplemental VitAD injection in sows during late gestation on circulating retinol and immune parameters in piglets. To do this, sows who were not clinically vitamin deficient were given a supplemental dose of VitAD on d 85 of gestation to determine if any improvements to production or health were observed in the offspring during the first 63 days of life. It was hypothesized that a supplemental injection of VitAD given to sows on d 85 of gestation would decrease the number of stillborn piglets, increase weight gain, increase retinol in serum and milk, and have no effect on immune parameters.

## Materials and methods

All animal procedures were approved by the Institutional Animal Care and Use Committee (IACUC: PROTO202101969) at The Pennsylvania State University.

Yorkshire (*n* = 9) and Yorkshire cross (*n* = 10) sows from the Penn State Swine Research Facility were randomly assigned to receive an intramuscular injection in the neck of 312,000 IU of vitamin A and 52,500 IU of vitamin D3 (administered in a volume of 0.624 mL) (VEDCO, Saint Josephs MO, USA) (*n* = 10), or 0.624 mL of saline (*n* = 9), on d 85 of gestation. Prior to administration of treatment, assessment of sow previous performance, parity, and genetic background was conducted to balance assigned treatments as necessary. At parturition, complete farrowing data was collected on all litters, including total number of piglets born, number of piglets born alive, stillborn piglets, and mummified fetuses. Necessary cross-fostering occurred within 12 hours of birth and all cross-fostering happened within treatment. All piglets were weighed daily from d 0 to 4, then weekly until d 63. During the preweaning phase (d 0—d 28), piglets were weighed in baskets attached to hanging scale (FishFun model DGL-B; Amazon). At weaning, pigs were moved to a temperature controlled, indoor nursery weaned into groups of 25 to 28 pigs, regardless of sow treatment, with free choice dry feeders and nipple water systems. Nursery phase weights were collected on a WayPig Litter Scale (Raytec, Ephrata, PA). Individual pig weights were collected for the duration of the study, from birth to the final weight collected during the nursery phase of production. Individual weights were possible, as all pigs on the research farm are individually identified by ear notches, and related back to litter of birth.

### Sow feed consumption

Gestating sows were given 5 lbs of feed per day and fed a diet adequate in VitAD according to the 2012 NRC requirements NRC 2012. Feed consumption was tracked during lactation for each sow using the Gestal Solo feeder (Jyga, St Lambert de Lauzon, QC, Canada). After farrowing, sows were given *ad libidum* feed until weaning. Using their nose, sows could lift a lever to trigger feed to drop and the total amount reported dropped in a day was used as a measure of feed consumption.

### Milk and offspring blood collection

At birth, umbilical cord blood was collected into a serum separator tube (Greiner Bio-One, Monroe, NC) prior to trimming and banding the umbilical cord on every other piglet born from each sow, with a maximum of 8 piglets per sow being selected for blood sampling. This subset of piglets had blood collected weekly via venipuncture from the jugular vein using a needle and syringe and stored in serum separator tubes (Greiner Bio-One, Monroe, NC) until d 63 of life to measure circulating retinol levels (d 0 to d 63) and immune parameters (d 28 to d 63). No more than 2 mL per piglet was collected each week.

Colostrum samples were taken at parturition, and subsequent milk samples were taken weekly until weaning (d 28). Colostrum and milk samples were collected by temporarily removing all piglets from the sow, utilizing supplementation with exogenous oxytocin (20 USP given as a 1 mL intramuscular injection (Bimeda Inc. Bimeda, Brazil) to stimulate milk let down, and hand extracted from all mammary glands in conical tubes (Avantor Inc., Radnor, PA) until at least 25 mL total was collected. Milk samples were kept on ice until processing, and no more than 80 mL of milk per sow was collected per collection day. Piglets were separated from the sows for no longer than 60 minutes. Blood and milk samples were protected from light to prevent retinol degradation and stored on ice until processing. Blood samples were processed by centrifugation for 15 minutes at 1940 x g at 10 °C and serum was transferred to individual microcentrifuge tubes and stored at −20 °C until analyzed. Milk samples were immediately stored at −20 °C.

### Immune parameter analysis

A CBC (Complete Blood Count) with a 5-part differential (white blood cell populations) was performed using a ProCyte Dx Hematology Analyzer (IDEXX Laboratories Inc. Westbrook, ME) by the Pennsylvania State University Centralized Biological Laboratory. Immune parameters assessed included hemoglobin (HGB), hematocrit (HCT), reticulocyte (RET), platelet (PLT), white blood cell (WBC), neutrophil (NEUT), lymphocyte (LYMPH), eosinophil (EO), basophil (BASO), monocyte (MONO), and red blood cell (RBC).

### Serum and milk retinol extraction process

All serum and milk samples were stored at −20 °C and protected from light during storage. Retinol was extracted from 100 µL of serum or milk as previously described (Ross 1986). In brief, retinol was saponified using 5% (w/w) potassium hydroxide in 95% ethanol, extracted in hexane, and reconstituted in 100 µL of methanol with 150 pmol trimethylmethoxyphenyl-retinol (TMMP-ROH) as an internal standard. To analyze retinol concentrations, 10 µL of lipid extracts in methanol were injected onto a *Waters Acquity* ultra-performance liquid chromatograph with a BEH C18 column (Waters Acquity: inner diameter: 2.1 mm, length: 50 mm, particle size: 1.7 µm) coupled with a *Waters Acquity* photo diode array (PDA) detector (Waters Corporation; Milford, MA). A constant flow of 92.5% methanol, and 7.5% deionized water was used with a flow rate of 0.6 mL/minute and a run time of 1.2 minutes. Retinol concentrations were analyzed by PDA detector using an absorbance wavelength of 325 nm with 4.8 nm resolution. Serum and milk retinol concentrations were calculated based on relative area counts of the internal standard, TMMP-ROH.

## Statistical analysis

All data was analyzed using PC SAS, (9.4; SAS, Cary, NC). All data was run using a mixed regression model (PROC MIXED); results were reported as least square means. Treatment was the main effect for all statistical analyses with a random effect of sow utilized to relate pigs back to litter throughout the growth phase. A Satterwaithe and Tukey-Kramer were applied to all analyses to account for unequal variance between litters and unequal sample sizes in the dataset. Mortality compared survival by treatment. For analysis of preweaning growth, parity, and litter size were included as secondary effects, and sow was included as a random effect. No differences were observed in parity of sows, which was assessed before sows received treatment and after treatment was randomly assigned.

Post-weaning growth data was analyzed as individual pig growth data and had days of age at weaning applied as secondary effects, with dam as a random effect. All body weight, growth, and ADG information was based on individual piglet performance and related back to sow for generating published least square means. Immune parameters used an interaction of date of sampling by treatment, with sow identification as a random. Feed consumption was evaluated by treatment. Lastly, milk and serum retinol was assessed with treatment applied as the primary effect. Descriptive statistics for offspring survival rate and sow performance parameters were evaluated using Microsoft Excel (Redmond, WA). Statistical differences were declared at *P *< 0.05, and tendencies were declared at *P *≤ 0.10.

## Results

### Fewer stillborn pigs were born to VitAD treated sows

Table 1 and Table S1 summarize the data collected describing litter performance, presented as least-square means of average piglet performance within the litter, at the time of farrowing. VitAD treatment did not alter the total number of piglets, piglets born alive, or number of mummified fetuses (*P *> 0.10). However, the VitAD litters had fewer stillborn offspring compared to control litters (*P *< 0.01). In total, 150 piglets were born in the control group (119 born alive) and 113 piglets in the VitAD group (106 born alive), as shown in Table S1 and Table 1. By day 28, 105 piglets survived in the control group and 88 in the VitAD group. Excluding stillbirths and mummified fetuses, the preweaning mortality rate was 16.5% in the control group and 17.9% in the VitAD group (*P *≥ 0.10).

**Table 1.** Litter performance at farrowing.

	Treatment			
Variable	Control	VitAD	SEM^a^	*P*-value
Total Born	15.3	12.9	1.38	0.22
Born Alive	12.7	11.8	1.19	0.58
Stillborn	1.4	0.3	0.28	**0.01**
Mummified Fetus	0.9	0.4	0.31	0.31
Mortality (%)	16.50	17.90	4.60	0.83

Total born, born alive, stillborn, and mummified fetus are reported as a count, and mortality is reported as a total percentage. Piglets were cross-fostered within treatment, pigs weaned were not different between treatments. Mortality was calculated without the inclusion of stillborn or mummified fetuses and is only a representation of death post survival at birth.

a Maximum standard error measurement (SEM) was used. Significant differences declared at *P *< 0.01.

### VitAD supplementation reduced sow feed consumption on day 15

**Fig. 1.**
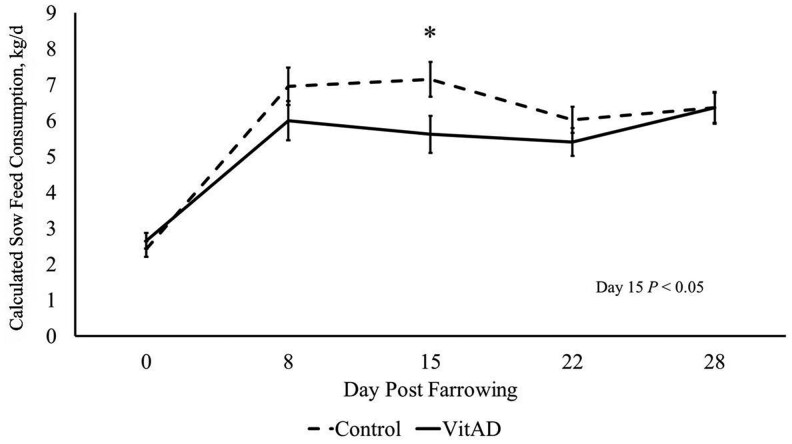
Sow feed consumption in the control and VitAD groups during the lactation phase. *N* = 10 control sows and *n* = 9 VitAD sows. Least square means ± SEM presented. Significant differences declared at *P < 0.05.

Feed consumption was monitored daily as the amount of feed dropped from each individual Gestal Solo per day. Figure 1 illustrates weekly sow feed consumption data from farrowing to d 28. At farrowing, feed consumption was not altered in response to VitAD supplementation (*P *> 0.10). On d 15 the VitAD sows triggered feed to drop considerably less than the control sows, consuming 5.62 kg compared to 7.15 kg of feed (*P *< 0.05). VitAD supplementation did not affect feed consumption at any other time point investigated (*P *> 0.10).

### VitAD sows had a greater concentration of retinol in milk on d 8

**Fig. 2.**
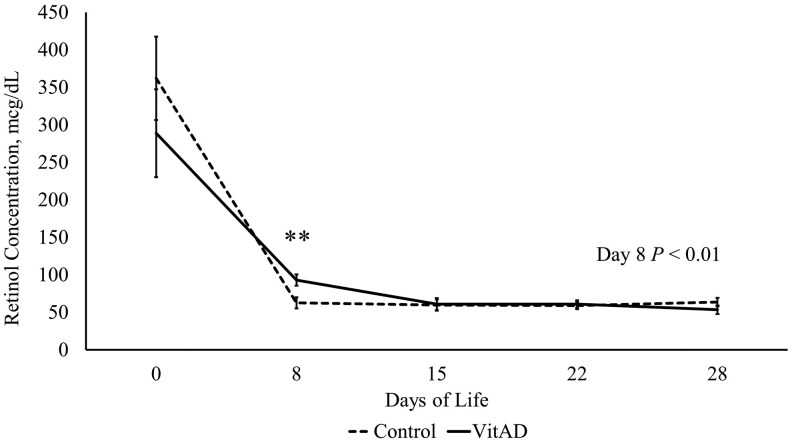
Retinol concentration in sow colostrum and milk during lactation. Least square means ± SEM presented. VitAD *n* = 45, control *n* = 48. Significant differences declared at **P < 0.01.

Temporal changes in milk retinol concentration are shown in Fig. 2. Milk retinol concentration on d 8 was greater in the VitAD supplemented sows (93.09 g/dL) compared to control sows (62.75 µg/dL) (*P *≤ 0.01). No significant differences in milk retinol levels were observed on other days evaluated (*P* > 0.10). No effect of VitAD supplementation on milk retinol concentration was detected on the other days investigated (*P *> 0.10).

### VitAD supplementation did not alter preweaning or postweaning growth

Pig weight and average daily gain (ADG) data are presented Tables 2 and 3, presented as least-square means of average individual pig weights, related back to birth sow. The VitAD piglets tended to have a reduced ADG between d 22 and d 28 (*P *= 0.08) compared to control piglets. No other statistical differences were observed in piglet weights or ADG during the pre- or post-weaning stages (*P *> 0.10). Similarly, there was no effect of VitAD treatment on pig weight or ADG from weaning through d 63 (*P *> 0.10), (Tables 2 and 3).

**Table 2.** VitAD Treatment did not alter piglet weights (within litter) from birth through d 63.

	Treatment			
Weight, kg	Control	VitAD	SEM^a^	*P*-value
Birthweight	1.21	1.19	0.08	0.87
d 2	1.26	1.33	0.11	0.59
d 3	1.38	1.41	0.10	0.81
d 4	1.56	1.48	0.12	0.58
d 8	2.27	2.21	0.16	0.74
d 15	3.83	3.59	0.25	0.42
d 22	5.36	4.90	0.34	0.25
d 28	7.58	7.02	0.41	0.27
d 35	8.63	8.14	0.43	0.37
d 42	11.26	10.95	0.56	0.66
d 49	14.47	14.50	0.71	0.97
d 56	18.58	18.56	0.87	0.99
d 63	22.67	22.69	0.99	0.98

Piglet weights. From birth weight—d 28 weights VitAD pigs (*n* = 113), control pigs (*n* = 150). From d 28—d 63 VitAD pigs (*n *= 55), control pigs (*n* = 71).

a Maximum standard error measurements (SEM) were used. No significant differences reported (*P *> 0.10).

**Table 3.** VitAD Treatment did not affect average daily gain of offspring within litter.

	Treatment			
Period	Control (kg/d)	VitAD (kg/d)	SEM^a^	*P*-value
Birth to d 4	0.11	0.06	0.04	0.28
d 4 to d 8	0.17	0.23	0.05	0.28
d 8 to d 15	1.58	1.38	0.13	0.19
d15 to d 22	0.22	0.18	0.02	0.17
d 22 to d 28	0.30	0.25	0.02	0.08^b^
Birth to d 28	0.22	0.19	0.02	0.12
d 28 to d 35	0.18	0.19	0.02	0.85
d 35 to d 42	0.38	0.40	0.03	0.44
d 42 to d 49	0.46	0.51	0.04	0.34
d 49 to d 56	0.59	0.58	0.03	0.87
d 56 to d 63	0.58	0.60	0.03	0.55

Table 4 depicts the ADG of the piglets in each treatment group between time points birth to d 28 VitAD pigs (*n* = 113), control pigs (*n* = 150). d 28 to d 63 VitAD pigs (*n* = 55), control pigs (*n* = 71).

a Maximum standard error measurements (SEM) were used.

b Tendency declared at (*P *≤ 0.10).

### VitAD supplementation resulted in lower offspring serum retinol concentration

**Fig. 3.**
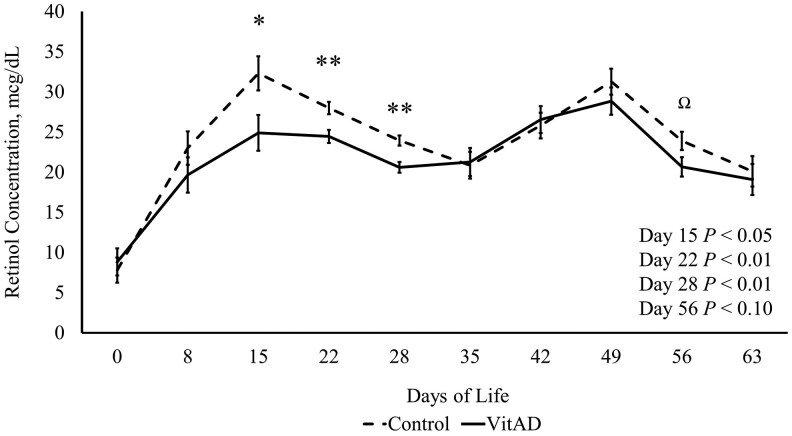
Piglet serum retinol concentration from birth to day 63. VitAD *n* = 86, control *n* = 95. Least square means ± SEM presented. Significant differences declared at *P < 0.05, **P < 0.01. ^Ω^Tendency declared at *P* ≤ 0.10.

Serum retinol concentrations were lower in VitAD offspring compared to control offspring on d 15 (*P *≤ 0.05), 22 (*P *≤ 0.01), 28 (*P *< 0.01) and 56 (*P *= 0.07; Fig. 3).

### Select immune parameters were temporally affected by VitAD treatment

**Fig. 4.**
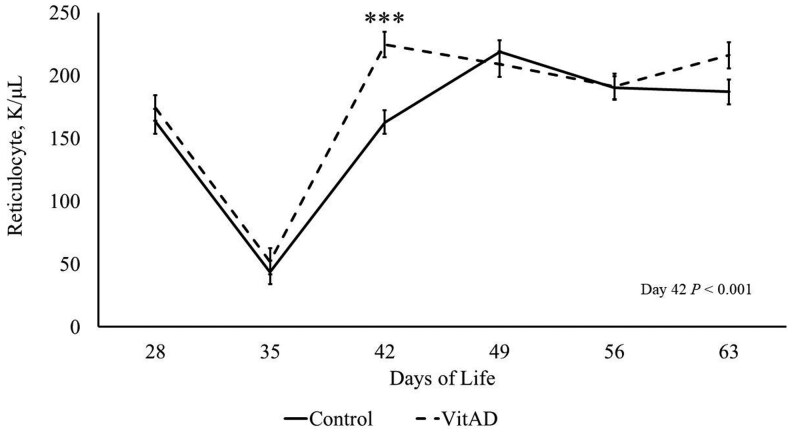
Piglet reticulocyte count from day 28 to day 63. VitAD *n* = 49 to 53, control *n* = 53 to 63. Least square means ± SEM presented. Significant differences declared at ***P < 0.001.

Immune parameters investigated on d 35 and 42 are presented in Table 4, while data for d 28, 49, 56, and 63 are included in Table S2. There was no effect of VitAD treatment on HGB, HCT, PLT, WBC, NEUT, EO, BASO, MONO, or RBC at any timepoint investigated (*P *> 0.10). Temporal and VitAD treatment effects are illustrated in Fig. 4. The VitAD pigs tended to have a higher RET count (*P *= 0.06) and on d 42, VitAD pigs had a greater RET count compared to control pigs (*P *≤ 0.01). This difference followed a low RET count on d 35 in both the control and VitAD group. VitAD pigs tended (*P* = 0.10) to have a lower LYMP count compared to control pigs on d 35.

**Table 4.** Immune parameters investigated on d 35 and d42 in selected pigs post weaning.

	D 35				D 42			
Immune Parameter	Control	VitAD	SEM^a^	*P*-Value	Control	VitAD	SEM^a^	P-Value
RBC, M/μL	6.56	6.47	0.08	1.00	6.11	6.05	0.08	1.00
RET, K/μL	43.53	52.08	10.46	1.00	162.77	224.59	10.22	**0.0006**
PLT, K/μL	337.91	392.81	27.45	0.94	562.25	519.48	27.13	0.99
WBC, K/μL	14.92	13.20	0.70	0.80	19.41	18.66	0.68	1.00
NEUT, K/μL	5.22	5.47	0.33	1.00	7.66	7.29	0.33	1.00
LYMPH, K/μL	8.54	6.65	0.46	0.10^b^	9.83	9.55	0.45	1.00
MONO, K/μL	1.01	0.93	0.08	1.00	1.68	1.59	0.08	1.00
EO, K/μL	0.13	0.12	0.02	1.00	0.22	0.20	0.02	1.00
BASO, K/μL	0.01	0.02	0.00	0.94	0.02	0.02	0.00	0.97
HCT, %	39.50	39.31	0.69	1.00	34.06	33.66	0.69	1.00
HGB, G/dL	11.04	10.95	0.15	1.00	9.58	9.63	0.15	1.00

Control and VitAD piglets are compared at every test date, with maximum standard error measurement used. Immune parameters assessed included hemoglobin (HGB), hematocrit (HCT), reticulocyte (RET), platelet (PLT), white blood cell (WBC), neutrophil (NEUT), lymphocyte (LYMPH), eosinophil (EO), basophil (BASO), monocyte (MONO), and red blood cell (RBC). VitAD *n* = 49 to 53, control *n* = 53 to 63.

a Maximum standard error measurement (SEM) was used. Significant differences declared at *P *≤ 0.05.

b Tendency declared at *P *≤ 0.10.

## Discussion

Over the past twenty years, sows have been genetically selected to become hyperprolific. Despite these substantial physiological changes, nutritional recommendations have not been reevaluated and it remains unclear if current dietary vitamin recommendations are sufficient to meet the nutritional requirements of the modern sow. Given ongoing debate surrounding the adequacy of current dietary vitamin recommendations, litter characteristics at the time of farrowing and piglet pre-weaning survival may serve as an indirect indicator of maternal vitamin sufficiency and health status. The objective of this study was to determine if supplementation of VitAD utilizing an approved injectable VitAD source on d 85 of gestation would improve litter characteristics at farrowing, offspring growth rates, immune parameters, and both milk and piglet circulating retinol concentrations.

The present study found that the number of stillborn offspring decreased in the VitAD group, compared to the control, which supports previous research that a supplemental injection reduces the incidence of stillborn offspring (Coffey and Britt 1993; Lindemann et al 2008). Similar results have been observed in human studies where mortality rates decreased in infants supplemented with vitamin A who were clinically deficient (Rahmathullah et al 1990; Daulaire et al 1992). In these cases, once sufficient standards were met, individuals were healthier or had an increased chance of survival compared to their placebo counterparts. These findings suggest that supplemental VitAD may have a role in the incidence or stillborn offspring, even in the absence of overt VitAD deficiency.

Postnatal piglet survival did not differ between groups, with mortality rates of 16.5% and 17.9% in the control and VitAD groups, respectively. VitAD sows had smaller total litter sizes (averaging 12.9 piglets vs. 15.3 piglets in controls). All sows enrolled in the study were evaluated for previous parity performance and all sows were bred to the same sire. Despite this, sows still had differences in the total number of pigs born per treatment. We do not believe these differences in litter size are due to the VitAD treatment, given that the injection was administered at d 85 of gestation, and no differences in mummified pigs were observed. While large litter sizes do tend to have more stillborn pigs than smaller litter sizes, which may have been contributing to the differences observed in this study, the trial was designed to account for these potential differences to the best of our ability. These results may suggest improved survival at birth in the VitAD group resulting in more piglets that survived the peri-natal period which warrants further investigation.

The two treatment groups of sows were fed identical diets during gestation and lactation periods respectively, but when injected with VitAD, there was an observed difference in how much feed the sows dropped in front of them. Sow feed consumption was tracked based on how much feed was dropped from the Gestal Solos. The control sows required more feed on d 15 of lactation, consuming 7.15 Kg of feed compared to 5.62 Kg in the VitAD sows. This difference in consumption was not accompanied by an effect on growth rates in the offspring, so the decreased intake of the VitAD group did not impact growth. Additionally, on d 15 the two groups had comparable numbers of surviving piglets. This lack of difference in litter size and weight gain compared to the decreased feed consumption needs to be further studied to investigate if this was the result of differences in litter size at birth, or insufficient ability to meet nutritional needs from the diet alone.

A single injection of vitamin A (312,000 IU) and vitamin D (50,000 IU) was administered to evaluate the impact on sow and piglet performance. The injection dosage is calculated based on the 30-day time period between injection and farrowing, with a goal to provide enough additional vitamin A to supply 2.5× the sows requirements during late gestation. The relatively large injection should be well tolerated, as a toxic dose of vitamin A in humans is 3000 times the recommended daily allowance (Allen and Haskell 2002), and a similar dose in sows would need to exceed 12,000,000 IU. Additionally, a single injection of up to 1,000,000 IU was observed to be well-tolerated by sows (Pusateri et al 1999). Injection of vitamin A was shown to persist for 210 days after injection (Perry et al 1967) while oral supplementation was shown to be less effective than injectable supplements in swine (Ullrey et al 1965), humans (Sommer et al 1980), and cattle (Knight and Death 1999). It was determined, based on these parameters, that an injection of 312,000 IU should not pose harm to the sows or piglets.

Vitamins A and D were not evaluated independently in favor of utilizing commercially available vitamin supplements. Currently, the only vitamin A injectables on the U.S. market approved for livestock that enter the food chain contain vitamin D, and often E as well. Vitamins, particularly A and D, share synergistic impacts on immune development. As lipid soluble vitamins, both vitamins are capable of being transported directly through the lymphatic system and have been observed to support the immune system, reproductive success, and for growth of offspring (Gropper et al 2017). Both Vitamin A and D have been observed to have impacts on inflammation through the production of T helper immune cells and B cells. Vitamin A is known to have morphogenic and functional impacts on fetal development, including ocular integrity, organ function, lung development, and skeletal growth during the third trimester (D’Ambrosio et al 2011; Youness et al 2022). Further, receptors for retinyl esters are known to be found in key immune system cells such as enterocytes in the small intestine, lung tissue, lymphatic system, fat depots, and in the skeletal muscle in rodents (Goodman et al 1965). Vitamin D supports the immune system directly and indirectly through driving development (Sîrbe et al 2022). Further, Vitamin D has also been observed to benefit immune response in the intestinal tract and in epithelial tissue, by reducing inflammatory response (Zhang et al 2012) and by modulating leukocytes and phagocytosis in wean pigs (Konowalchuk et al 2013). These similar pathways make products like injectable vitamin A and D a practical tool for swine producers, should the supplement be found to have similar impacts under production conditions.

The focus on this study was to evaluate any potential benefits to the piglets because of sow supplementation. The immune parameters investigated were chosen based on a pilot study (Hines, unpublished) where piglets were injected with VitA Immediately following birth, and a reduction in white blood cell count, lymphocytes, monocytes, and basophils were observed to have decreased by d 42 of age among piglets that recieved the vitamin A injection at birth. Similar results were not observed in the current study, rather, when sows were injected at d 85 of gestation, no differences in immune parameters were observed, with the exception of a difference in RET concentration on d 42 with the VitAD offspring having an increased (224.56 K/μL) concentration as compared to the control offspring (162.77 K/μL). An immature RBC, RET measurements indicated a stage of blood cell replacement that have just been released from the bone marrow (Anovadia et al 2023). Once matured, RBC carry with their functional hemoglobin (Anovadia et al 2023). As previously discussed, both vitamin A and vitamin D have been observed to modulate the immune system. However, those previous studies did not show an association with RET cells specifically. This novel finding should be further investigated as it may relate to piglet health under commercial conditions, potentially in relationship to anemia in piglets (Jiang et al 2009). Currently, standardized immune reference ranges for swine across developmental stages are lacking A previous study has reported RET values on d 30 as ∼148.2 10^9^ cell/L, with a range of 53.4 to 554.5 10^9^ cell/L (Ventrella et al 2017). RET counts in this study generally fell within that range, except on day 35, when values were lower (43.53 and 52.08 K/μL for control and VitAD groups, respectively), potentially reflecting a transient post-weaning stress response. The low RET count on d 35 could in turn result in decreased RBC, HCT, and HGB on d 42, but this was not observed. However, IDEXX reports that RET in pigs should never exceed 100 K/μL at any age (IDEXX 2019), which would make the offspring in this study within the correct range on d 35 but elevated for the remainder of the study period. Similar reference ranges have been reported in the Merck Veterinary Manual, with slight differences in suggested MONO and RET counts (Merck 2023). Of the existing swine immune parameter investigations and reference ranges, preferred units for reporting findings have not been established resulting in potential confusion when comparing results. Most other swine investigations that include hematology do not include RET count, so these results will help improve the current knowledge about what normal RET is in piglets. These discrepancies highlight the need for more comprehensive reference data throughout stages of production. This study provides important insights into temporal dynamics in immune parameters and supports broader efforts to define age-appropriate hematologic norms in pigs. There have been several investigations and reported reference ranges suggesting what is considered normal for immune parameters in pigs (Ventrella et al 2017; IDEXX 2019; Merck 2023), but there is variation within the data, and reference criteria are poorly understood. The data collected from the current study will contribute to the understanding of what are considered normal immune parameters in weaned piglets and how they may be affected by interventions.

Serum retinol measurements were taken weekly from birth until the end of the nursery phase (d 63) to examine if an injection of VitAD given to sows had any effect on circulating retinol level in the offspring. Interestingly, supplementing sows with VitAD on d 85 of gestation resulted in lower serum retinol for offspring through much of the preweaning and once during the post weaning phase of growth (Fig. 3). This reduction in serum retinol was unexpected; however, it does fall within the published normal range for serum retinol. Past evaluations have reported serum retinol ranging from 12 to 30 µg/dL in 10 to 42-day old pigs (Surles et al 2007; Vlasova et al 2013; Gannon et al 2017), and 5 to 20 µg/dL in piglets 0 to 25 hours old (Gannon et al 2017). The current study found similar results with the piglets having an average of 22.81 µg/dL regardless of treatment. On d 15, the serum retinol concentration spiked for control pigs, reaching a maximum of 44.67 µg/dL in some pigs. It is unknown as to why the VitAD offspring had lower retinol concentrations as compared to the control offspring through d 28 of life. It should be noted that the current study is the first study to evaluate serum retinol in piglets when all sows are fed a diet sufficient in vitamin A, according to current NRC recommendations. Normal circulating retinol in pigs of any age have not been established. It has previously been observed that pigs between 10 and 42 days of age can have circulating retinol between 12 and 30 µg/dL (Surles et al 2007; Vlasova et al 2013; Gannon et al 2017). In contrast, retinol concentrations in newborn piglets have been reported at 5 µg/dL from 0 to 4 hours old, and 6-20 µg/dL from 5 to 25 hours old (Gannon et al 2017). However, these studies fed sows deficient diets prior to farrowing so serum retinol concentrations might not reflect normal serum retinol concentrations in piglets. While similar experiments evaluating the use of injectable vitamin A have been conducted in the past, the data collected on serum retinol and immune parameters in piglets from sows in this study can contribute to the body of work needed to support evaluations of modern animal nutritional needs and immune status.

Retinol concentrations in milk were quantified to evaluate potential differences in vitamin A availability to piglets. Vitamin A has low bioavailability to pigs, predominantly due to limited sources in the common corn and soybean-based diet (Ullrey 1972). To overcome this, most pig diets are supplemented with a form of vitamin A, however final uptake of the supplement is considered to vary. A pig’s exposure to retinol concentration is thus decreased as it ages, as Vitamin A is naturally the highest in colostrum, followed by a quick drop off as it transitions to milk (Macias and Schweigert 2001). The colostrum collected in the current study contained 362.15 and 288.9 µg/dL of retinol in the control and VitAD group, respectively. The retinol concentration decreased significantly between d 0 and 8, with a slope of −37.4 µg/day compared to −24.5 µg/day in the control and VitAD groups, respectively. This could suggest the supplementation of VitAD helped extend the elevated retinol concentration past d 0, which is similar in human studies that gave a dose of vitamin A to mothers and improved colostrum retinol concentration (Grilo et al 2015). As pigs age and transition further from colostrum and milk as primary sources of fat-soluble vitamins, their body stores and nutritional requirements may fluctuate, and potentially impact the development of offspring. Findings from this work suggest that more investigation is needed into the impact of gestational nutrition on milk content.

In the Penn State herd, the Yorkshire sows are not similarly productive to the hyperprolific lines observed in industry, where the average litter size is closer to 14 than it is to the current national average (PIC 2017). The Penn State Yorkshires, however, like many genetic lines, have been selected for improved litter sizes over decades, and have an average litter size of 11 pigs, similar to the improved national average. Further, being a University herd, the sows and piglets are generally healthier than commercial pigs. Additionally, given the relatively low sample size in this study, future investigations into the potential of this supplementation should utilize larger groups of sows to demonstrate that these findings translate to larger herd sizes. Given that we observed these interesting findings in our university farm settings, we believe this warrants further investigation under commercial conditions to test the results of this study under field challenges and opportunities.

The results of this study support the body of available research that indicates that supplemental injection of vitamins improves litter performance, with the data indicating that an injection of VitAD helps to decrease the number of stillborn piglets. This research also supports that growth rate, sow milk retinol, and sow feed consumption were not negatively affected by the treatment, and no other potential negative side effects were observed. This paper is also the first to report weekly retinol concentration in pigs at birth through the first 63 days of life, and weekly post weaning immune parameters including how weaning effects concentration. While research on this topic continues to be conflicting, we believe more research needs to be conducted to improve the current understanding of normal retinol concentrations in pigs of varying stages of production, including if vitamins have a persistent benefit on immune parameters.

## Supplementary Material

txaf134_Supplementary_Data

## List of abbreviations:

25OHD3: 25-hydroxycholecalciferol (vitamin D)
ADG: Average Daily Gain
BASO: Basophil
BW: Birth Weight
CBC: Complete Blood Count
d: Day
EO: Eosinophil
HCT: Hematocrit
HGB: Hemoglobin
LYMPH: Lymphocyte
MONO: Monocyte
NEUT: Neutrophil
PDA: Photo Diode Array
PLT: Platelet
RBC: Red Blood Cell
RET: Reticulocyte
TMMP-ROH: Trimethylmethoxyphenyl-retinol
VitAD: Vitamin A and D
WBC: White Blood Cell
