# Supplementary material for: The effect of late gestation injectable vitamin A and D supplementation on sow and piglet performance
Source: Transl Anim Sci. 2025 Sep 30;9:txaf134. doi: 10.1093/tas/txaf134 (PMC12659805; doi:10.1093/tas/txaf134)
Supplement: txaf134_Supplementary_Data [file txaf134_supplementary_data.zip › Supplemental Table 1.docx]

**Supplemental Table 1: Descriptive Statistics for Individual Litter Performance at Farrowing**

| **Sow** | **Treatment** | **Parity** | **Total Born** | **Born Alive** | **Stillborn** | **Mummified Fetuses** |
| --- | --- | --- | --- | --- | --- | --- |
| 1 | Control | 3 | 14 | 11 | 2 | 1 |
| 2 | Control | 3 | 10 | 8 | 2 | 0 |
| 3 | Control | 3 | 12 | 10 | 2 | 0 |
| 4 | Control | 3 | 14 | 12 | 1 | 1 |
| 5 | Control | 4 | 22 | 19 | 2 | 1 |
| 6 | Control | 1 | 17 | 15 | 1 | 1 |
| 7 | Control | 1 | 17 | 13 | 1 | 3 |
| 8 | Control | 2 | 15 | 12 | 3 | 0 |
| 9 | Control | 1 | 12 | 11 | 0 | 1 |
| 10 | Control | 0 | 17 | 16 | 0 | 1 |
| 11 | VitAD | 3 | 18 | 18 | 0 | 0 |
| 12 | VitAD | 1 | 12 | 9 | 0 | 3 |
| 13 | VitAD | 1 | 16 | 15 | 1 | 0 |
| 14 | VitAD | 3 | 17 | 15 | 2 | 0 |
| 15 | VitAD | 2 | 7 | 7 | 0 | 0 |
| 16 | VitAD | 3 | 11 | 11 | 0 | 0 |
| 17 | VitAD | 1 | 14 | 13 | 0 | 1 |
| 18 | VitAD | 1 | 12 | 12 | 0 | 0 |
| 19 | VitAD | 3 | 6 | 6 | 0 | 0 |
