# Supplementary material for: The effect of late gestation injectable vitamin A and D supplementation on sow and piglet performance
Source: Transl Anim Sci. 2025 Sep 30;9:txaf134. doi: 10.1093/tas/txaf134 (PMC12659805; doi:10.1093/tas/txaf134)
Supplement: txaf134_Supplementary_Data [file txaf134_supplementary_data.zip › Supplemental Table 2.docx]

**Supplemental Table 2: Immune parameters investigated on d 28, 35, 42, 49, 56, and 63**

|  | D 28 | | | | D 35 | | | | D 42 | | | | | | |
| --- | --- | --- | --- | --- | --- | --- | --- | --- | --- | --- | --- | --- | --- | --- | --- |
| Immune Parameter | Control | VitAD | SEM^1^ | P-Value | Control | VitAD | SEM^1^ | P-Value | Control | VitAD | | SEM1 | | P-Value | |
| RBC, M/μL | 6.47 | 6.44 | 0.08 | 1 | 6.56 | 6.47 | 0.08 | 1 | 6.11 | 6.05 | | 0.08 | | 1 | |
| RET, K/μL | 163.44 | 174.16 | 10.13 | 1 | 43.53 | 52.08 | 10.46 | 1 | 162.77 | 224.59 | | 10.22 | | **0.001***** | |
| PLT, K/μL | 547.38 | 507.5 | 27.01 | 1 | 337.91 | 392.81 | 27.45 | 0.94 | 562.25 | 519.48 | | 27.13 | | 0.99 | |
| WBC, K/μL | 11.33 | 11.25 | 0.68 | 1 | 14.92 | 13.2 | 0.7 | 0.8 | 19.41 | 18.66 | | 0.68 | | 1 | |
| NEUT, K/μL | 3.39 | 3.3 | 0.32 | 1 | 5.22 | 5.47 | 0.33 | 1 | 7.66 | 7.29 | | 0.33 | | 1 | |
| LYMPH, K/μL | 6.85 | 6.93 | 0.45 | 1 | 8.54 | 6.65 | 0.46 | 0.10 ^Ω^ | 9.83 | 9.55 | | 0.45 | | 1 | |
| MONO, K/μL | 0.89 | 0.83 | 0.08 | 1 | 1.01 | 0.93 | 0.08 | 1 | 1.68 | 1.59 | | 0.08 | | 1 | |
| EO, K/μL | 0.18 | 0.18 | 0.02 | 1 | 0.13 | 0.12 | 0.02 | 1 | 0.22 | 0.2 | | 0.02 | | 1 | |
| BASO, K/μL | 0.01 | 0.01 | 0 | 1 | 0.01 | 0.02 | 0 | 0.94 | 0.02 | 0.02 | | 0 | | 0.97 | |
| HCT, % | 35.33 | 36.28 | 0.68 | 1 | 39.5 | 39.31 | 0.69 | 1 | 34.06 | 33.66 | | 0.69 | | 1 | |
| HGB, G/dL | 10.45 | 10.73 | 0.15 | 0.96 | 11.04 | 10.95 | 0.15 | 1 | 9.58 | 9.63 | | 0.15 | | 1 | |
|  | D 49 | | | | D 56 | | | | D 63 | | | | | | |
| Immune Parameter | Control | VitAD | SEM^1^ | P-Value | Control | VitAD | SEM^1^ | P-Value | Control | | VitAD | | SEM^1^ | | P-Value |
| RBC, M/μL | 6.41 | 6.36 | 0.08 | 1 | 6.38 | 6.49 | 0.08 | 1 | 6.55 | | 6.55 | | 0.08 | | 1 |
| RET, K/μL | 218.74 | 209.17 | 10.14 | 1 | 190.22 | 191.35 | 10.14 | 1 | 186.93 | | 216 | | 10.29 | | 0.67 |
| PLT, K/μL | 395.02 | 378.93 | 27.03 | 1 | 331.81 | 345.44 | 27.03 | 1 | 396.46 | | 378.54 | | 27.23 | | 1 |
| WBC, K/μL | 18.28 | 16.16 | 0.68 | 0.48 | 18.08 | 19.53 | 0.68 | 0.92 | 18.72 | | 18.6 | | 0.69 | | 1 |
| NEUT, K/μL | 7.03 | 6.5 | 0.32 | 0.99 | 5.96 | 5.84 | 0.32 | 1 | 5.38 | | 5.27 | | 0.33 | | 1 |
| LYMPH, K/μL | 9.59 | 8.07 | 0.45 | 0.34 | 10.08 | 11.51 | 0.45 | 0.45 | 11.39 | | 11.46 | | 0.45 | | 1 |
| MONO, K/μL | 1.45 | 1.41 | 0.08 | 1 | 1.86 | 2 | 0.08 | 0.98 | 1.72 | | 1.66 | | 0.08 | | 1 |
| EO, K/μL | 0.18 | 0.17 | 0.02 | 1 | 0.16 | 0.16 | 0.02 | 1 | 0.23 | | 0.19 | | 0.02 | | 1 |
| BASO, K/μL | 0.01 | 0.02 | 0 | 1 | 0.02 | 0.02 | 0 | 1 | 0.01 | | 0.01 | | 0 | | 1 |
| HCT, % | 36.36 | 35.91 | 0.68 | 1 | 36.91 | 37.73 | 0.68 | 1 | 37.93 | | 37.62 | | 0.69 | | 1 |
| HGB, G/dL | 10.5 | 10.4 | 0.15 | 1 | 10.56 | 10.72 | 0.15 | 1 | 10.97 | | 10.93 | | 0.15 | | 1 |

Control and VitAD piglets are compared at every test date, with maximum standard error measurement used. Immune parameters assessed included hemoglobin (HGB), hematocrit (HCT), reticulocyte (RET), platelet (PLT), white blood cell (WBC), neutrophil (NEUT), lymphocyte (LYMPH), eosinophil (EO), basophil (BASO), monocyte (MONO), and red blood cell (RBC). VitAD n= 49-53, control n= 53-63. ^1^Maximum standard error measurement (SEM) was used. Significant differences declared at *P* ≤ 0.05. Ω Tendency declared at *P* ≤ 0.10.
